# The relationship between physical activity and premenstrual syndrome in senior high school students: a prospective study

**DOI:** 10.1038/s41598-023-32357-2

**Published:** 2023-04-11

**Authors:** Zhuo Chen, Kazuhiro Imai, Xiao Zhou

**Affiliations:** 1 Department of Life Sciences, Graduate School of Arts and Sciences, The University of Tokyo, 3-8-1, Komaba, Meguro-ku, Tokyo, 153-8902 Japan; 2 School of Physical Education of Huazhong University of Science and Technology, Wuhan, China

**Keywords:** Epidemiology, Paediatric research

## Abstract

Reproductive-aged women experience somatic and affective symptoms of premenstrual syndrome (PMS) which affect their daily life and work, but there are limited tracking data on senior high school-age female students. To investigate the characteristics and incidence of PMS, and to detect the relationship between physical exercise participation and PMS in female students of senior high school. A prospective study was conducted among senior high school female students aged 14–16 years. The participant was asked to finish two questionnaires. One questionnaire collected information about demographic data and PMS symptoms which were recorded on a daily calendar named Daily Record of Severity of Problems (DRSP) per day. Another questionnaire was physical exercises participation questionnaire, collecting times of physical education (PE) class participation, exercise times, exercise type participation in morning exercise and recess exercise, times of morning exercise, times of recess exercise per week, recess exercise time, and autonomous exercise participation of students. The data were prospectively recorded for three consecutive months. Odds ratio (OR) with 95% confidence intervals (CI) was performed for results of the multivariate logistic regression analysis model. A total of 233 participants completed the prospective study, 78 (33.5%) participants experienced PMS. The incidence of the participants with mild PMS was 20.2% while moderate PMS was 11.6% and severe PMS was 1.7%. The most common somatic symptom was fatigue while the most common affective symptom was cannot concentrate. Participants with PE classes less than 2 times per week were 4.43 times (OR 4.43, 95% CI 1.18–16.6, *p* < 0.05) more possible to experience PMS than those with 2 times PE classes per week. PMS is a frequent phenomenon in senior high school female students. Female students with 2 times PE classes per week show a lower incidence of PMS. This study encouraged senior high school-age females to take more physical exercises per week and could be helpful to create a no-pharmacology coping strategy.

## Introduction

Premenstrual syndrome (PMS) is a generic term that many reproductive women feel physical or mood changes during the days before menstruation and subsides following the menstrual period, which affects a woman’s normal life^1^. In the premenstrual phase of the menstrual cycle, symptoms of PMS are classified as mild, moderate, and severe. Women who suffer from severe PMS similar to those with major depression will interfere with their normal relationships in school, and social and family activities.

The prevalence of PMS is not consistent in different areas and different places of the same state. A study showed that the incidence of PMS was 21.1% in Chinese adult women^2^, whilst the prevalence of Japanese adult women was 5.3%^3^. A systematic review with meta-analysis study showed that more than half of the women under reproductive age group experienced PMS in Ethiopia^4^. Moreover, the incidence of mild PMS was 11.3–93.5%, moderate was 4.6–5.3%, and severe was 1.2–2.1%^2,5^. More than 90% of women over the age of 18 experienced at least one PMS symptom. Fatigue or lack of energy was the most common somatic symptom whereas the most common affective symptom was irritability^6^. There were 20% of adult women reported that PMS significantly disturbed their way of daily life^7^.

The prevalence of PMS in adolescents was 10–64.6% which presents significantly inconsistent^8^. Among all the symptoms of PMS, angry outbursts and breast tenderness were the most common affective and the most common somatic symptoms, respectively. Moreover, a retrospective study showed that the incidence of moderate and severe PMS in Japanese senior high school female students was higher than Japanese adult women^6^. The senior high period is unique in human development both psychologically and physiologically, which is an important and vulnerable period through the lifespan^8^. In retrospective design studies about PMS, it was likely to have recall bias influenced the results. Nonetheless, epidemiological studies of PMS in adolescents were not well understood, especially with a prospective study design.

As for PMS prevention, the American College of Obstetricians and Gynecologists recommends physical exercises to prevent the occurrence of PMS^9^. Literature reported Yoga, moderate-intensity aerobic exercise to be effective in reducing the incidence of PMS among females aged over 18 years^10^. However, as far as we have researched, no prospective studies detecting the association between physical exercises and PMS in senior high school students were found. The purpose of this study was to investigate the characteristics and incidence of PMS and to identify the association between physical exercises participation and PMS in senior high school female students.

## Methods

This study collaborated with the Yingxiongshan senior high school of Shandong province in China. This research protocol was obtained from the Graduated School of Arts and Sciences at the University of Tokyo. The research protocol was approved by Ethical Committee of the Graduate School of Arts and Sciences, the University of Tokyo, Japan (Notification Number 602-2 July 26, 2018). The study protocol was performed in compliance with the tenets of the Declaration of Helsinki. An informed written consent form had been signed by each female student and their guardians before participation in the study.

### Participants and questionnaires

A school-based survey conducted from November 2019 to January 2020 for three consecutive months. We recruited 699 female students aged 14–16 years in Taian Yingxiongshan senior high of Shandong province, an east city of China. Before tracking, 403 participants were selected from all 699 participants, using the eligibility criteria as follows: (1) regular menstrual cycles (25–35 days), (2) no mental illness in the past 2 years, (3) being able to provide information consent. After 3 consecutive months tracking, targeted participants were excluded, if they: (1) incomplete data, (2) used hormonal medication, (3) drunk alcohol in the 3 months. Finally, 233 participants completed the prospective study.

Two paper questionnaires were used to collect data, consisting of basic parameters, PMS, and physical exercise participation investigation. The Daily Record of Severity of Problems Chinese version provides reliable and valid measure of premenstrual symptoms in Chinese women^14^, the physical exercise questionnaire was sent to four professionals for assessment verification. An acculturated pilot study was conducted to improve the questionnaire quality. The Cronbach alpha value of this questionnaire was 0.814, it had highly reliable.

To investigate and diagnose PMS, we applied the validated scale Daily Record of Severity of Problems (DRSP) which is the most accepted and widely used system to diagnose PMS. The participants needed to fill it day-to-day for at least two consecutive menstrual cycles^11–13^. We send the DRSP at the beginning of every month, and the participants were asked to fill the DRSP at the end of per day; at the end of the month, the questionnaires were collected; then the DRSP of the next month was sent. Symptoms (10 total terms) of PMS in the DRSP consisted of affective symptoms and somatic symptoms. The affective symptoms included 5 items, that is depression, feeling out of control, irritability, anxiety, and cannot concentrate. The somatic symptoms included the other 5 items, that is breast tenderness, gastrointestinal symptoms, fatigue, headache, and bloating. In this study, the diagnosis of PMS was made according to the diagnostic criteria proposed by the American College of Obstetricians and Gynecologists (ACOG). The criteria of each item used the points of 0–3, that is 0 was no symptom, 1 was the mild symptom, 2 was the moderate symptom (affecting their daily study and life but tolerable), and 3 was severe symptom (seriously affecting their daily study^15^). Participants recorded the severity of the symptoms per day using the criteria. Also, they recorded the day of their menstrual for 3 months. PMS was diagnosed if one or more met the screening criteria: (1) one or more of the items that assess depression, anxiety, feeling out of control, and irritability has scores of at least 4 for 2 days before menses; (2) the participant has scores of at least 4 for at least 2 days before menses. If the participant does not have any symptoms that judged as no PMS.

**Figure 1:**
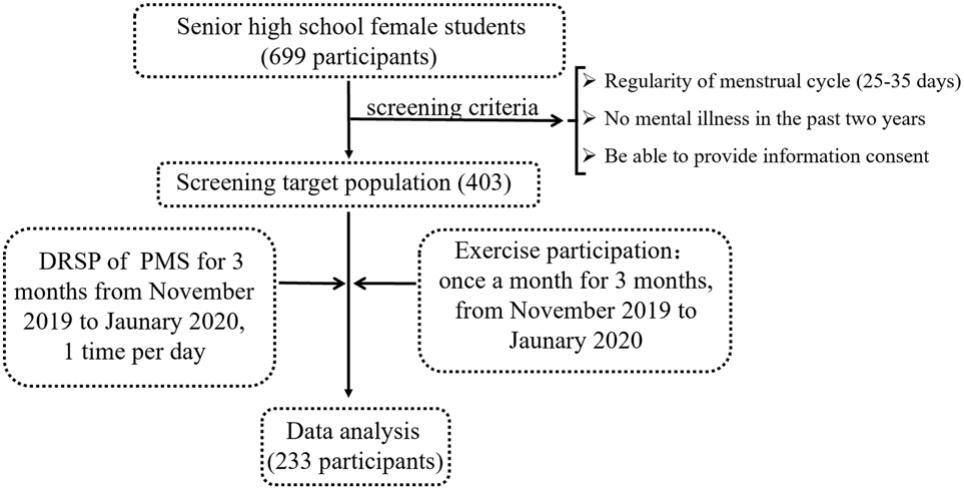
Flowchart of study participants.

The physical exercises participation questionnaire consisted of four parts. The first part consisted of the times of PE class per week, physical exercise types and minutes per PE class. The second part included the times of morning exercise and recess exercise per week, the minutes of morning and recess exercise per time. The third part included the times of autonomous exercise participation per week, minutes, type and forms of autonomous exercise participation per time. The last part included the exercise participation attitudes consisting of higher willingness, general willingness and unwillingness. At the end of the month, the physical exercise participation questionnaires were sent to the participants for recording their exercise condition per month of three consecutive months. The eligible criteria are shown in Fig. 1.

### Statistical analysis

All statistical analysis was performed using SPSS for Windows Version 26.0 (IBM Corp). The normality of basic parameters (i.e., age, height, weight, body mass index (BMI, kg/m^2^) calculated by dividing weight by height squared, exercise time per week, minutes per day and total minutes per week) was tested via Shapiro–Wilk test. Due to the normal distribution of the data, a student *t*-test was operated to compare basic parameters between the groups. Descriptive statistics including mean ± SD or percentage were used for describing demographic information and the incidence of PMS. Medians with interquartile range (IQR) were admitted to present continuous variables that were categorized based on the distribution. The association between the presence of PMS and participants’ exercise condition was analyzed via the binary logistic and multivariate logistic regression analysis. A 95% CI of the prevalence of PMS was calculated to determine the preciseness of the primary outcome. Statistical significance was set at *p* < 0.05.

## Results

According to the result of DRSP, we found that among 233 participants, 78 (33.5%) participants who experienced PMS were divided into PMS group, and the rest 155 (66.5%) participants without PMS were divided into no PMS group. Basic parameters of the two groups are shown in Table 1. There were no significant differences of characteristics between the PMS group and the no PMS group.

**Table 1:** General information for female students of senior high school.

Characteristics	PMS (n = 78)	No-PMS (n = 155)
Age (year)	15.6 ± 0.7	15.7 ± 0.8
Height (cm)	164.1 ± 5.0	163.5 ± 5.1
Weight (kg)	57.3 ± 8.7	56.2 ± 9.3
BMI (kg/m^2^)	21.2 ± 2.9	20.98 ± 3.0
PE class/week		
≤ 1	25 (32.1)	17 (11.0)
2	53 (68.9)	138 (89.0)
Minutes per class		
< 10	24 (30.8)	20 (12.9)
10–20	37 (47.4)	99 (63.9)
20–45	17 (21.8)	36 (23.2)
Morning exercise times per week		
≤ 4	6 (7.7)	5 (3.2)
5	72 (92.3)	150 (96.8)
Minutes per morning exercise		
0–10	1 (1.3)	2 (1.3)
10–20	33 (42.3)	63 (40.6)
20–30	44 (56.4)	90 (58.1)
Recess exercise per week		
≤ 4	2 (2.6)	2 (1.3)
5	76 (97.4)	153 (98.7)
Minutes per recess exercise		
0–10	33 (42.3)	66 (42.6)
10–20	43 (55.1)	85 (54.8)
20–30	2 (2.6)	4 (2.6)

Data are presented as number (percentage) or mean ± SD.

*BMI* body mass index, *PMS* premenstrual syndrome.

**Figure 2:**
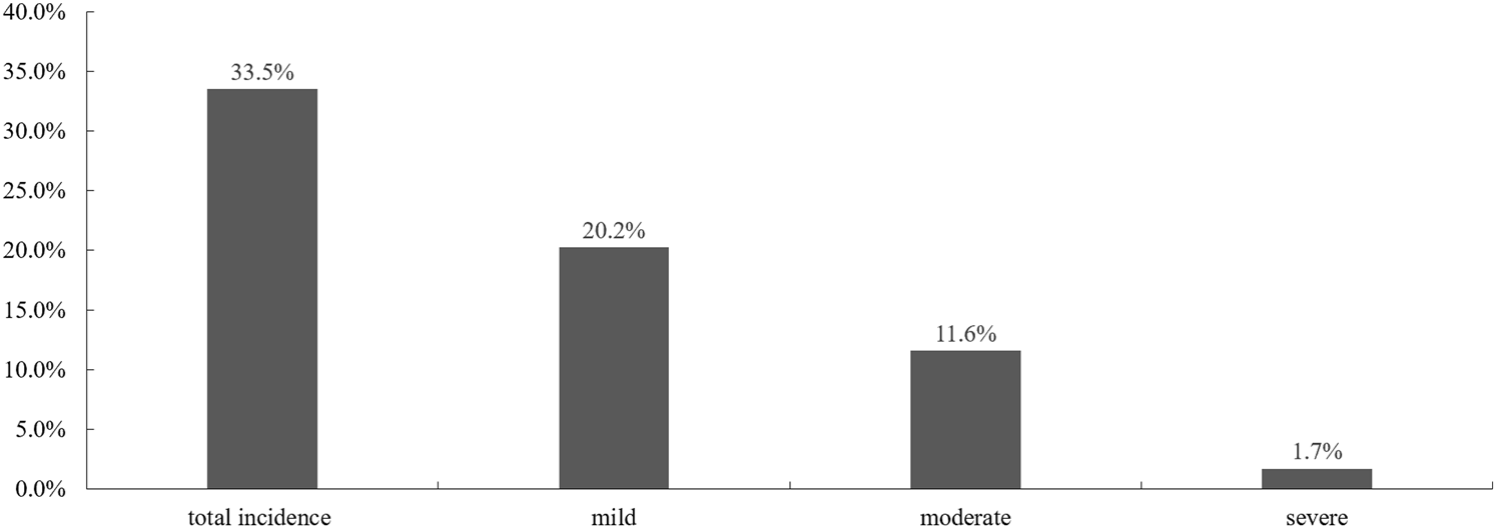
The incidence of PMS in female students of senior high school.

The prevalence of PMS in senior high school was 33.5%. Among all the participants, the prevalence of the female students with the mild PMS was 20.2% while those with the moderate PMS was 11.6% and those with the severe PMS was 1.7% (Fig. 2).

**Figure 3:**
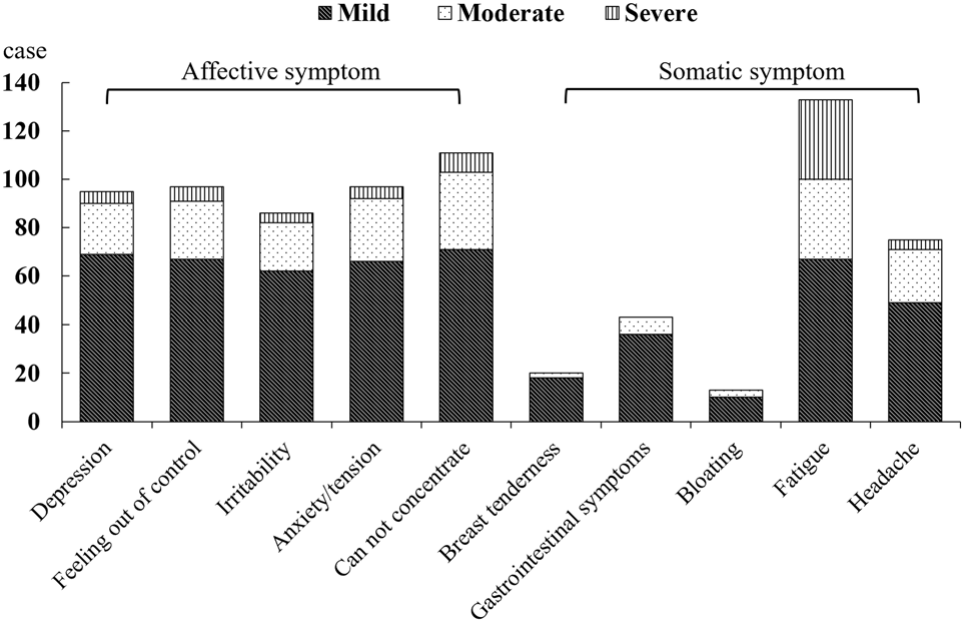
Distribution of PMS symptoms in female students of senior high school.

Results of the distribution of symptoms of PMS are shown in Fig. 3. The total symptoms were 770 cases, including 486 case affective symptoms and 284 case somatic symptoms. The most common somatic symptom was fatigue, 46.8% (133 cases: 67 mild cases, 33 moderate cases, and 33 severe cases) of all the somatic symptoms, followed by headache (26.4%/75 cases; 49 mild cases, 22 moderate cases, and 4 severe cases), gastrointestinal symptoms (15.1%/43 cases, 36 mild cases, 7 moderate cases, and no severe case), breast tenderness (7.0%/20 cases; 18 mild cases, 2 moderate cases and no severe case) and bloating (4.6%/13 cases; 10 mild cases, 3 moderate cases and no severe case). As for affective symptoms, the most common affective symptom was cannot concentrate, 22.8% (111 cases; 71 mild cases, 32 moderate cases, and 8 severe cases) of all the affective symptoms, followed by depression (19.5%/95 cases; 69 mild cases, 21 moderate cases, and 5 severe cases), feeling out of control (20.0%/97 cases; 67 mild cases, 24 moderate cases, and 6 severe cases), anxiety (20.0%/97 cases; 66 mild cases, 26 moderate cases, and 5 severe cases) and irritability (17.7%/86 cases; 62 mild cases, 20 moderate cases, and 4 severe cases).

The association between each variable and the PMS was analyzed by binary logistic regression analyses that the presence of PMS significantly associated with PE class times per week and minutes of per PE class (Table 2). The participants with PE class ≤ 1 were 3.38 times (OR 3.38, 95% CI 1.92–7.66, *p* < 0.01) more likely to sustain PMS than those with 2 times PE classes per week. The participants with exercise times less than 10 min were 2.54 times (OR 2.54, 95% CI 1.11–5.81, *p* < 0.05) more likely to sustain PMS than those with exercise times more than 10 min per class.

**Table 2:** Binary logistic regression analyses of variables associated with PMS.

Variables	N (%)	PMS, N (%)	OR (95% CI)	*p* value
PE class, per week				
2	191 (82.0)	53 (67.9)	1.00	
≤ 1	42 (13.3)	25 (32.1)	3.83 (1.92–7.66)	< 0.001
Minutes, per PE class				
20–45	44 (18.9)	17 (21.8)	1.00	
10–20	136 (58.4)	37 (47.4)	0.79 (0.40–1.58)	n.s
< 10	53 (22.7)	24 (30.8)	2.54 (1.11–5.81)	0.032

*OR* odds ratio, *CI* confidence intervals, *PE* physical education, *PMS* premenstrual syndrome.

Multivariate logistic regression analysis was used to adjust for age, BMI, minutes per PE class, times of autonomous activities, minutes per autonomous activity, days of morning exercises per week, minutes per morning exercise, times of recess exercises per week, minutes of per recess exercise and exercise participation attitude. Results of the multivariate logistic regression analysis are shown in Table 3. The PMS was significantly associated with PE class less than 1 time per week (OR 4.43, 95% CI 1.2–16.6, *p* < 0.05).

**Table 3:** Multivariate logistic regression analysis of variables associated with PMS.

Variable	N (%)	PMS, N (%)	Adjusted, OR (95% CI)	*P* value
PE class, per week				
2	191 (82.0)	53 (67.9)	1.00	
≤ 1	42 (13.3)	25 (32.1)	4.43 (1.18–16.60)	0.001

*PMS* premenstrual syndrome, *OR* odds ratio, *CI* confidence intervals, *PE* physical education.

## Discussion

We attempted to identify the epidemiology of PMS, and the association between the occurrence of PMS and physical exercise participation in senior high school female students. This study provided unique findings as follows: (1) with the 3 months tracking, the incidence of PMS in senior high school female students was 33.5% of all the participants, including 20.2% mild PMS, 11.6% moderate PMS, and 1.7% severe PMS, (2) the most common somatic symptom was fatigue and the most common affective symptom was cannot concentrate, (3) senior high school female students with PE class less than 2 times per week showed higher incidence of PMS.

Regarding the prevalence of PMS in senior high school female students, a prospective study of Thai high school students showed that the incidence of PMS was 29.8%. The most common somatic symptom was angry outbursts, and the most common affective symptom was breast tenderness^16^. A retrospective study of Japan showed that 64.6% Japanese senior high school female students experienced PMS, fatigue was the most common somatic symptom while anxiety was the most common affective symptom^17^. The incidence of PMS in female students in our prospective studies was similar to Thailand, it was 33.5%. The most common somatic symptom was fatigue that in line with Japanese senior high school students^6^. In addition, 91% of our participants with PMS experienced affective symptom of cannot concentrate that different from previous studies^16,18^. Different cultures of China such as dietary habits and life customs, and data collection methods used might cause the different incidence of PMS across these studies^19^.

Additionally, the prevalence of PMS in Chinese women aged 18–45 was 21.1%, the most emotional symptom was irritability, and the most common physical symptom was breast tenderness and abdominal bloating^2^. The incidence of PMS in senior high school female students in the current study was higher than Chinese adult women. The most common somatic symptom and affective symptom were different from Chinese adult women. The causes of the difference in the incidence of PMS between adolescent and adult women were unknown. We suspected that female students in senior high school suffer from undergoing tremendous physical and psychological changes on their way to adulthood. In addition, they regularly experience stress associated with studies as well as their sexual and fruitful health^17^.

Although the causes of PMS have not been clearly elucidated, however, it has been suspected to include abnormal concentrations of sex steroids^20^, serotonin, and γ-aminobutyric acid (GABA)^21^. Sex steroid production provokes luteal symptoms that were triggered by fluctuations of such hormones^8^. The participants of our studies were puberty adolescent females whose hormone is instability and increases with age. These findings also support our study that the incidence of PMS in senior high school female students was higher than adult women.

With regard to the association between PMS and exercise participation, some studies revealed that aerobic exercise, such as yoga, swimming, treadmill exercise and stretch, and resistant exercise could reduce PMS^22^. Yoga could regulate the autonomic nervous system (CNS) and increase the activity of the parasympathetic nervous system (PNS) and γ-aminobutyric acid (GABA) system^21^. When participants were swimming, nervous tension would be changed and mediate PMS by altering neurotransmitters such as endorphins^23,24^. Treadmill exercise could raise serum progesterone levels^7^, improve and release endorphins^25^. Resistant exercise and stretching had a reduction in stress levels and release endorphin anxiety^26^. Moreover, stretch-induced estrogen enhanced the expression of glycometabolism-related enzymes and proteins, helping regulate glucose metabolism^27^. In sum, numerous previous studies expressed a useful role of physical exercise in maintaining hippocampal hormonal levels^3,10,24^, and consequently hippocampal malleability, knowledge, and mood regulation that might reduce PMS symptoms^3^.

Physical education curriculum of Chinese senior high school, comprising of preparation activities, sports skills learning (long jump, high jump, etc.), jogging, 800-m race, sit-ups, 50-m race, stretching, jumping rope and autonomous exercise, focuses on health promotion and regular physical-exercises participation in China^28^. There are 2 lessons per week, 45 min for each lesson. Meanwhile, one morning exercise and two recess exercises are performed per day. In this study, participants took some aerobic exercise weekly, such as jogging, 800-m stretching, and rope skipping. These findings support our results that the age of 15–17 years old female students with more PE class times per week had lower incidence of PMS than those without. Moreover, previous studies revealed that exercise habits of more than 30 min twice a week may reduce the severity of premenstrual symptoms in the women aged 18–25 years^29^. The results indicated that physical exercises could be effective in reducing the incidence and severity of PMS in women aged 15–25. However, a previous study which is inconsistent with our studies revealed that there is no significant association between physical exercises and PMS^25^. The previous study is a cross-sectional study which causes the recall bias that might be interpreted the differences.

There are some limitations in the current study. First, self-rating of PMS symptoms could be somewhat difficult for female students in senior high school students, which may increase concerns about the reliability of the data. Second, PMS affects school absenteeism in Japanese female high school students^17^, however, in this study, we have not investigated the participants whether PE class absence or not caused by PMS that might cause the association between PMS and PE class per week. Third, waist circumference^30^, age at menarche, sleeping hours, eating habit, and pain killers which might affect the incidence and severity of PMS of senior high school female students that are supposed to be studied in future. Final, the notably high prevalence and the association between physical exercises and PMS in Chinese senior high school in this study warrant future large-scale study to evaluate the impact of PMS on their quality of life, academic performance, and effective intervention for mitigating PMS among senior high school female students.

## Conclusion

The new and unique findings of this study are that PMS is a common phenomenon among Chinese senior high school female students that the incidence of PMS in senior high school students was 33.5%. The most common somatic symptom and affective symptoms were fatigue and cannot concentrate, respectively. The female students with 2 times PE class per week show a lower incidence of PMS than those without. Based on the findings, our study suggested that physical exercises might reduce PMS symptoms, facilitate health promotion for female senior high school students and could help to develop a non-pharmacology coping strategy.

## Data Availability

The datasets used and/or analyzed during the current study available from the corresponding author on reasonable request.
